# Occluded Grape Cluster Detection and Vine Canopy Visualisation Using an Ultrasonic Phased Array

**DOI:** 10.3390/s21062182

**Published:** 2021-03-20

**Authors:** Baden Parr, Mathew Legg, Stuart Bradley, Fakhrul Alam

**Affiliations:** 1 Department of Mechanical and Electrical Engineering, Massey University, 229 Dairy Flat Highway, Auckland 0632, New Zealand; b.parr@massey.ac.nz (B.P.); f.alam@massey.ac.nz (F.A.); 2 Inverse Acoustics Ltd., 2 Rata Street, Auckland 0600, New Zealand; inverse.acoustics@gmail.com

**Keywords:** ultrasound, array, vine yield, canopy estimation, smart agriculture, nondestructive, remote sensing

## Abstract

Grape yield estimation has traditionally been performed using manual techniques. However, these tend to be labour intensive and can be inaccurate. Computer vision techniques have therefore been developed for automated grape yield estimation. However, errors occur when grapes are occluded by leaves, other bunches, etc. Synthetic aperture radar has been investigated to allow imaging through leaves to detect occluded grapes. However, such equipment can be expensive. This paper investigates the potential for using ultrasound to image through leaves and identify occluded grapes. A highly directional low frequency ultrasonic array composed of ultrasonic air-coupled transducers and microphones is used to image grapes through leaves. A fan is used to help differentiate between ultrasonic reflections from grapes and leaves. Improved resolution and detail are achieved with chirp excitation waveforms and near-field focusing of the array. The overestimation in grape volume estimation using ultrasound reduced from 222% to 112% compared to the 3D scan obtained using photogrammetry or from 56% to 2.5% compared to a convex hull of this 3D scan. This also has the added benefit of producing more accurate canopy volume estimations which are important for common precision viticulture management processes such as variable rate applications.

## 1. Introduction

The ability to accurately estimation grape yield is important because it allows viticulturist to plan, increase profitability, and improve the quality of the grapes produced. Yield estimation allows viticulturists to implement precision agriculture techniques including crop thinning, variable rate applications, and selective harvesting [1]. Traditionally, manual processes are used to estimate yield such as visual inspection and cutting and weighing grapes within a section of the vineyard [2]. However, these manual processes can be time consuming and the generally low number of samples taken can lead to inaccurate estimations. There is a need for an automated technique to accurately estimate grape yield.

Computer vision techniques have therefore been developed for automatically counting the number of grapes visible in camera images, and a high accuracy has been reported [3]. However, one limitation is that these techniques rely on being able to see the grapes. Errors in grape yield estimation occur where grapes are occluded by leaves or other grape bunches [4]. This has been addressed by assuming a certain percentage of grapes are occluded and compensating using a scaling factor [5]. However, this is not ideal and can lead to errors. Another approach is to remove leaves from the grape vines which could cause occlusions [6,7]. However, this can be laborious unless specialised machinery is available. In addition, we understand that there are grape verities such as Gewürztraminer where foliage is normally not removed. Occlusion is perhaps the most significant unsolved issue for yield estimation using computer vision solutions.

One solution that has been suggested to address the issue of occlusion is microwave-based yield estimation [8]. The high frequency radio waves are able to propagate through foliage and reflect off the grape clusters behind. However, these devices are expensive and are not near commercial implementation. In this paper, we explore a previously unexplored alternative technology, ultrasound, for image through the leaves and detecting occluded grape bunches.

Within the field of precision viticulture, there have been several studies that have used ultrasound to map the outer leaf canopy shape for improved vineyard management. Gil et al. used three ultrasonic sensors to independently measure the distance to the vine foliage from spray nozzles positioned at different heights [9]. These transducers were positioned vertically in a line (tens of cm apart) and were operated independently to measure the distance to the foliage at three different heights. They were not used as an array. The closest distance reported by each sensor was used in real-time to control the application flow-rate from the nozzles. The benefit of this approach was verified by Llorens et al. who established that an average of 58% saving of application volume was obtainable [10]. In addition to variable rate application, independent scans taken over the growing season have been reported to have the potential to be an effective approach to monitoring vine vigour [11]. However, the effectiveness of these studies was limited by their use of ultrasonic transducers, which operated independently and not as arrays, to measure the distance to the outer surface of the foliage. These individual transducers have had a relatively wide beamwidth, and generally, the only information used from the reflected signal is the time of first echo from the foliage [11]. This results in low resolution imaging of the grapevine outer canopy and can give an overestimation of the canopy volume due to a few outer leaves sticking out [12]. Further work by Llorens et al. compared the same ultrasonic canopy measurements to a colocated 2D Light Detection and Ranging (LIDAR) scanner, a common alternative approach [13]. They found that the precise directionality of the laser distance measurements resulted in significant improvement in canopy surface estimation, albeit at the cost of a more complicated postprocessing procedure [14]. This highlights the utility that narrower beam-width ultrasonic sensors may offer.

**Figure 1:**
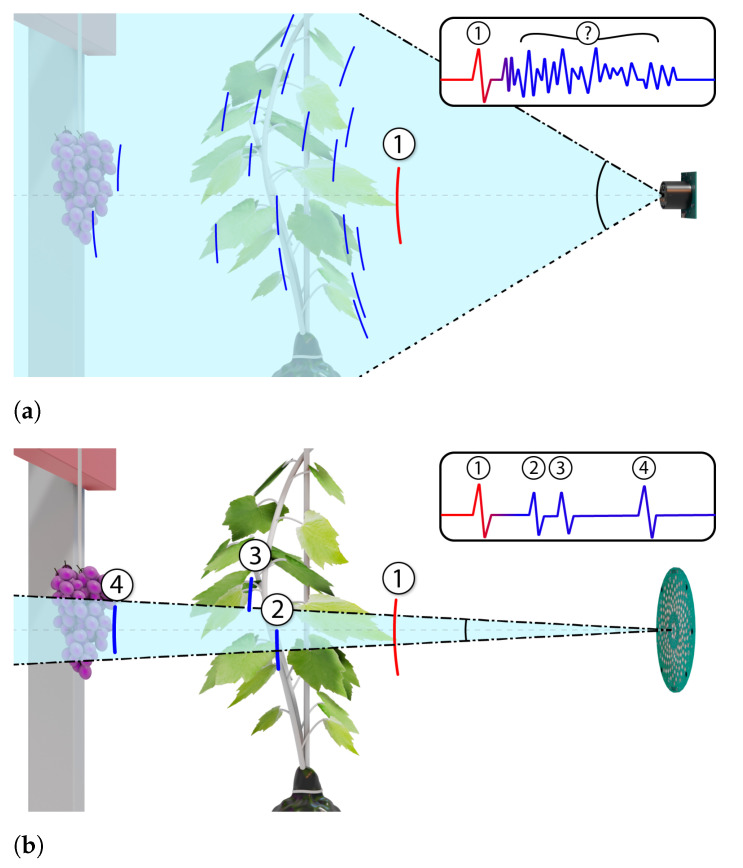
A single ultrasonic transducer (not an array) with a wide beamwidth can struggle to image objects behind the front leaves, as illustrates in diagram (**a**). In contrast, diagram (**b**) illustrates how an ultrasonic array such as used in this work (see Figure 2 for a photo) with a narrow beamwidth can provide improved ability to image at multiple depths behind the front leaves.

Recent work by Palleja et al. utilised four ultrasonic transducers to generate a volumetric estimation of a vine canopy using the signal envelope of multiple echoes [15,16]. In a similar manner to Gil et al. [9], these transducers were arranged vertically in a line with each transducer being spaced 45 cm apart. They were not used as an array but as four transducers operating independently. However, the transducers employed had a wide beam pattern and therefore poor imaging resolution. For busy scenes, an independent ultrasonic transducer will be sensitive to multiple echoes from objects in a wide field of view. This is beneficial for applications such as a car reversing system where the system is only interested in the distance to the closest object. However, for an imaging system where one wants to image through leaves, using a single ultrasonic transducer will result in poor angular resolution. This is not desirable as it will make it hard to detect structure behind the closest leaf, see Figure 1a. Traditionally, one might increase the directionality of ultrasonic transmission by using transducers which are operational at high ultrasonic frequencies (several hundred kHz). However, we anticipate that this would come at the expense of reduced penetration through foliage and increased attenuation. These difficulties may explain why no previous studies have been found in the literature that have used ultrasound to image fruit occluded by leaves.

Arrays of ultrasonic transducers can be used to increase angular resolution [17,18]. Figure 1b shows how an array of ultrasonic transducers can achieve a higher angular resolution compared with a single transducer. This significantly improves the potential for imaging structure behind the outer leaves. However, no previous study has been found which has used ultrasonic arrays to image any type of foliage apart from the authors’ work with pasture in references [19,20].

In this study, we present the first work where an ultrasonic array has been used to image grapes and foliage. To achieve an adequate angular resolution at lower ultrasonic frequencies (<60 kHz), we have utilised a novel air-coupled ultrasonic array developed by the authors [19,20]. Another issue with using low ultrasonic frequencies is the low depth resolution due to the large wavelengths and ringing of the transducers [21]. This has been addressed in this work using coded waveforms, cross-correlation, and operating away from the transducers’ resonant frequency. Ultrasonic arrays and coded waveforms have not been used before in precision viticulture.

The high spatial and depth resolution from the array allowed the echoes from grapes and leaves to be separated. However, the ultrasonic echoes from leaves and grapes appeared to be identical. This was addressed by making multiple ultrasonic measurements at the same location while lightly agitating the leaves with a fan directed at the measurement area. Since the leaves moved while the heavier grapes remained stationary, the mean and variance the ultrasonic measurements could be used to identify the grape bunch.

Initially, imaging was performed with the array focused in the far-field. Work was then undertaken to investigate the improvement in imaging resolution using near-field focusing of the array. This includes a novel technique to compensate the cross-correlation for near-field defocusing of the transmitted signal. The improved spatial resolution in the resulting volumetric scans will be a benefit for precision viticulture management processes such as variable rate applications where an accurate understanding of the vine canopy is vital.

This paper has the following significant contributions to knowledge. It is the first work to use an air-coupled ultrasonic phased array and coded waveforms for the purpose of analysing vine canopies. It is also the first study to investigate if it is possible to use ultrasound to image through leaves, to detect fruit located behind leaves, and to differentiate echoes that come from leaves through agitation. In addition, we present a new technique for improving the resolution of the array based cross-correlation for near-field echoes. This approach simulates the effect of focusing the transmission of the array at any desired depth in postprocessing. This eliminates the need for the complex electronics required for focusing the array’s transmission to a desired scan depth. Some preliminary results of this work were presented in the conference paper [22].

The paper is organised as follows. Section 2 introduces the ultrasonic array hardware and measurement parameters used in this work. The experimental setup and measurement procedure are described in Section 3. The signal processing applied to the array data for imaging grapes is then presented in Section 4. Section 5 and Section 6 provide results for the array focused in the far-field and near-field respectively. Finally, in Section 7, we end the paper with some final points and a discussion about future directions that can be taken.

## 2. Ultrasonic Array

**Figure 2:**
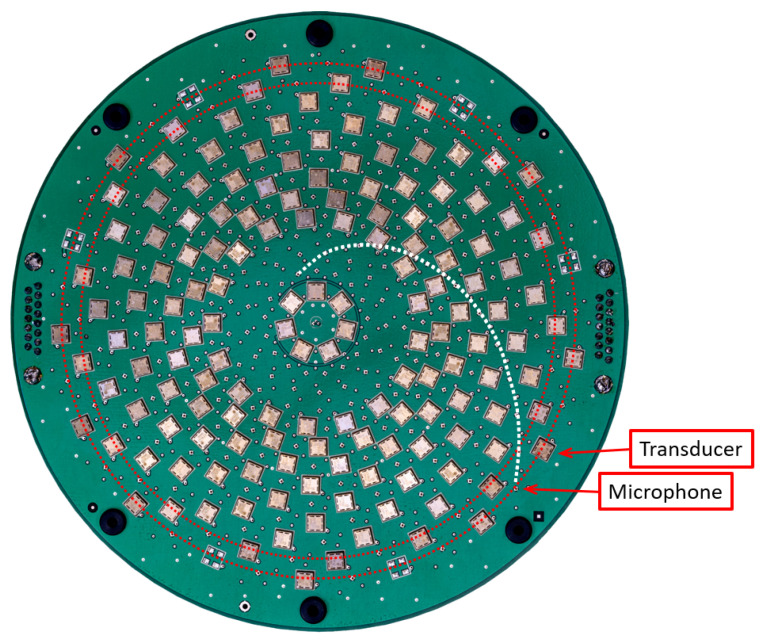
Photo of the ultrasonic array’s main PCB (printed circuit board). The transducers (silver squares) and microphones (located behind holes) are arranged in a multiarm spiral pattern forming rings. One of the microphone spirals arms is illustrated by a white dashed line while the two outer rings of microphones and transducers are shown as red dashed lines.

Figure 2 shows the ultrasonic array thas has been used in this work. This was custom designed and built by the authors for precision agriculture requirements. A full description of this array is given in reference [19]. It has optimised spiral arrays of 160 ultrasonic transducers and 204 microphones, which are arranged into rings. The transducers are surface mounted to the front of the array PCB. In contrast, the MEMS microphones (which can operate at ultrasonic frequencies) are surface mounted to the back of the PCB with holes passing though the PCB to allow the acoustic signal to be measured. The radius of the transducer and microphone rings are given in Table 1.

The microphone array had 12 independent rings of microphones. All the microphones in a ring were connected in parallel and then captured by one of 12 simultaneous sampling Analogue to Digital Converter (ADC) channels of a Data Translation DT9836 module [23], refer to Figure 12b in reference [19]. A sampling rate of 225 kHz and a resolution of 16 bits were used. Note that since all 12 microphone ring channels were saved to file, it was possible to dynamically change the focus distance of the reception in postprocessing using beamforming.

**Figure 3:**
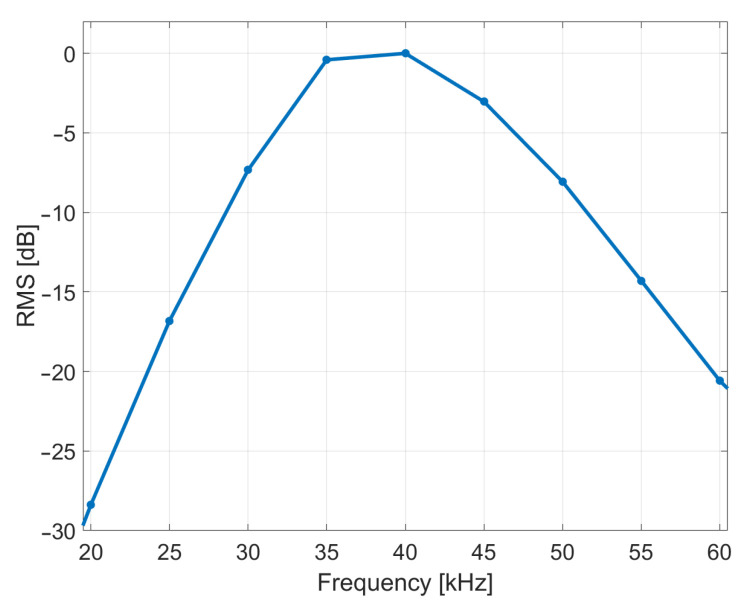
The surface mount transducers frequency response that was measured using the microphone model that was used in the array.

The transducers used in the array were surface mount air-coupled transducers which had a resonance frequency of 40 kHz and a frequency response which dropped from this peak by about 20 dB at 25 kHz and 60 kHz on either side. The measured frequency response can be seen in Figure 3. Although the transmission gain is highest around 40 kHz, the transducers have a tenancy to ring at this resonance frequency, which is undesirable if cross-correlation is being used to improved depth resolution. We therefore operate them at frequency ranges on either side of the resonant peak (e.g., 20–35 kHz and 45–60 kHz). The transducers were arranged in 10 rings. The DT9836 board’s two Digital to Analogue (DAC) channels were used to drive the 10 rings (half of the rings for each DAC channel) through two power amplifiers, refer to Figure 12a in reference [19]. These had an output sampling rate of 500 kHz and resolution of 16 bit and were synced with the ADC channels. Data acquisition software was written in MATLAB to transmit the signal and capture the resulting echoes using the DT9836 board.

**Figure 4:**
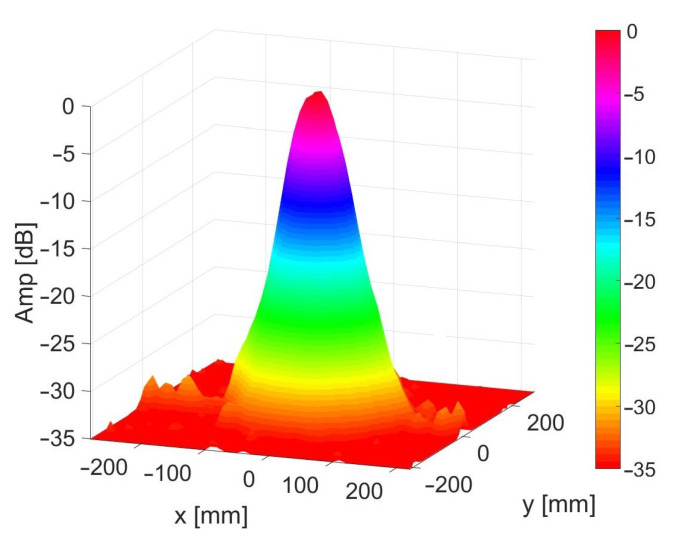
Plot of the measured array beam pattern (combining transmission and reception) for a 35 kHz sine wave using a 40 mm diameter reflector at 805 mm distance from the array.

The same excitation signal (a linear chirp) was applied to all the transducers. Since the array was planar (on a flat PCB), this meant that it was effectively using far-field beamforming with the transmission focused at a point in front of the array at infinity. Near-field focusing was not possible for transmission since we did not have a separate DAC channel controlling each transducer ring. Figure 4 shows the measured combined transmit/receive beam pattern of the array when the array is focused at infinity. This shows a full beamwidth of 3.3${}^{\circ }$ and a dynamic range of up to 33 dB. Please refer to reference [19] for details on how this beam pattern was obtained. The array had a dead-zone of about 500 mm where the signal measured by the receiver channels was dominated by the vibrations caused by the ultrasonic transmission. Objects closer than this were hard to detect.

Air-coupled transducers generally achieve a high gain at the expense of ringing at the resonant frequency of the transducer. As a result, digital codes such as Barker Codes or Maximum Length Sequence (MLS) with sharp transitions can cause ringing and may not be reproduced correctly by the transducers. In contrast, the lack of sharp temporal transitions for a chirp waveform means that it is less prone to exciting ringing of the transducer compared with some other waveforms.

The transmit linear chirp signal applied to the transducers can be described by

(1) $$y\left[n\right]=W\left[n\right]\cdot \sin\left(2\pi \left[f_{0}\;\;t\left[n\right]+\frac{\beta }{2}\;\frac{t{\left[n\right]}^{2}}{\tau }\right]\right),$$

where t[n] is the time of the $n_{th}$ transmit sample, $f_{0}$ is the start frequency at t=0, β is the bandwidth, τ is the pulse duration, and W is a Hamming window [24].

**Figure 5:**
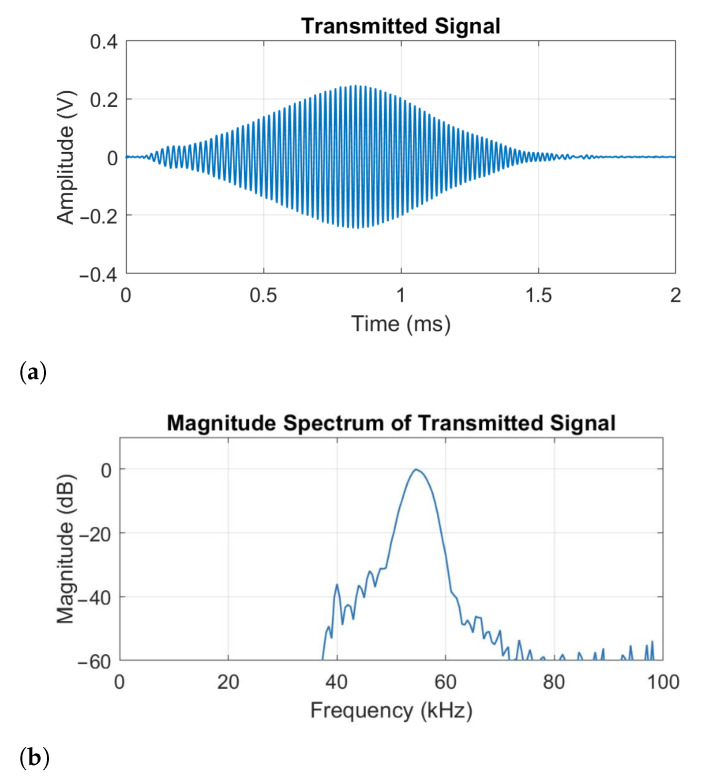
Plots showing (**a**) the ultrasonic chirp transmit signal as recorded by a calibrated microphone (GRAS 46BF-1 1/4 inch), and (**b**) its corresponding frequency domain representation. The small peak seen at 40 kHz is a result of ringing at the transducers’ fundamental frequency.

A chirp excitation signal was chosen as it can be used with cross-correlation to improve the depth resolution. After testing, a linear chirp with a duration of 1.5 ms and bandwidth of 45 to 60 kHz was chosen. This transmitted signal was verified through independent recording using a calibrated microphone (GRAS 46BF-1 1/4 inch). The signal time and frequency domain representations can be seen in Figure 5. The small time delay is due to the separation between the transducer and microphone. The 1.5 ms duration chirp appeared to provide improved cross-correlation resolution compared to a shorter duration chirp. The frequency bandwidth was chosen based on the frequency response of the transducers, which have a usable frequency range between 25 kHz and 60 kHz and a resonant frequency of 40 kHz, see Figure 3. To avoid ringing at this resonant frequency, the chirp used a frequency range from 45 to 60 kHz. It was also felt that this frequency range gave slightly better depth resolution than the 25–35 kHz range due to the smaller wavelength. The attenuation experienced by the ultrasound as it travels through the air can be calculated using the atmospheric absorption model given by International Standard ISO 9613-1:1993 [25]. It can be shown that at a standard atmospheric pressure, a temperature of 20 ${}^{{}^{\circ}}$C and 50% humidity at 60 kHz this is about 1.98 dB/m. For practical operation in a vineyard, the width of the rows limits the operating distance to about 1 meter. Over this distance, the attenuation is negligible.

## 3. Experimental Set-Up and Procedure

**Figure 6:**
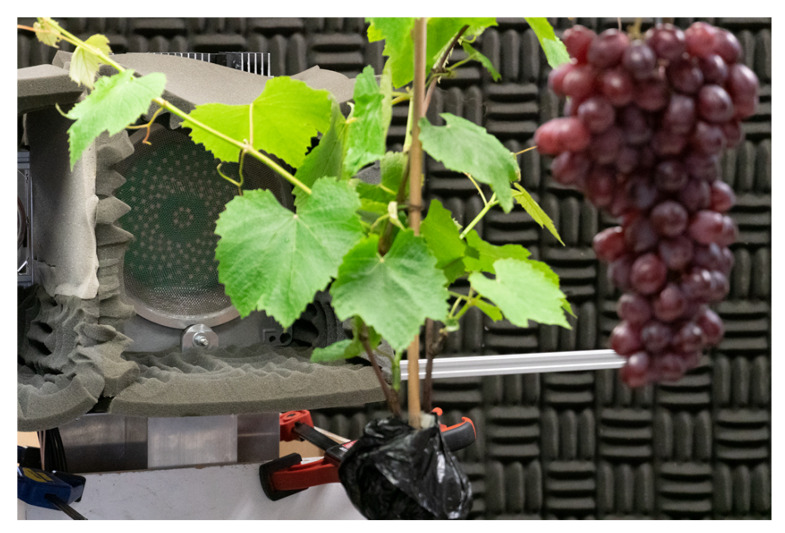
Photo showing the experimental setup with the grapes located behind a vine that is attached to a Computer Numerical Controlled (CNC) machine.

The ability of the array for detecting grapes was evaluated using a 2D Computer Numerical Controlled (CNC) gantry system. This CNC had a range of motion of 1.4 × 1.4 m and a resolution of 0.025 mm. Ideally the array would have been mounted to the CNC machine. However, the array was originally designed for operation from a farm vehicle and was too heavy in its current mounting. Instead a grape vine was mounted directly to the CNC machine. The grapes were fixed to 3 mm rods. This was done to reduce the amount of movement when the CNC was moving and to minimise reflections from this support. The vine was mounted to a bamboo pole and its roots were surrounded by a plastic bag with most of the soil removed to reduce weight. Refer to Figure 6 for a photo of the setup.

Initially acoustic foam had been placed behind the CNC to dampen echoes from the wall behind, as shown in Figure 6. However, subsequent measurements were made with the foam removed and no noticeable difference in measurement performance was observed. This was expected as the array has a highly directional beamwidth and as a result is very insensitive to reflections outside its field of view, as shown in Figure 4. This shows that in a field environment such precautions would not be necessary.

**Figure 7:**
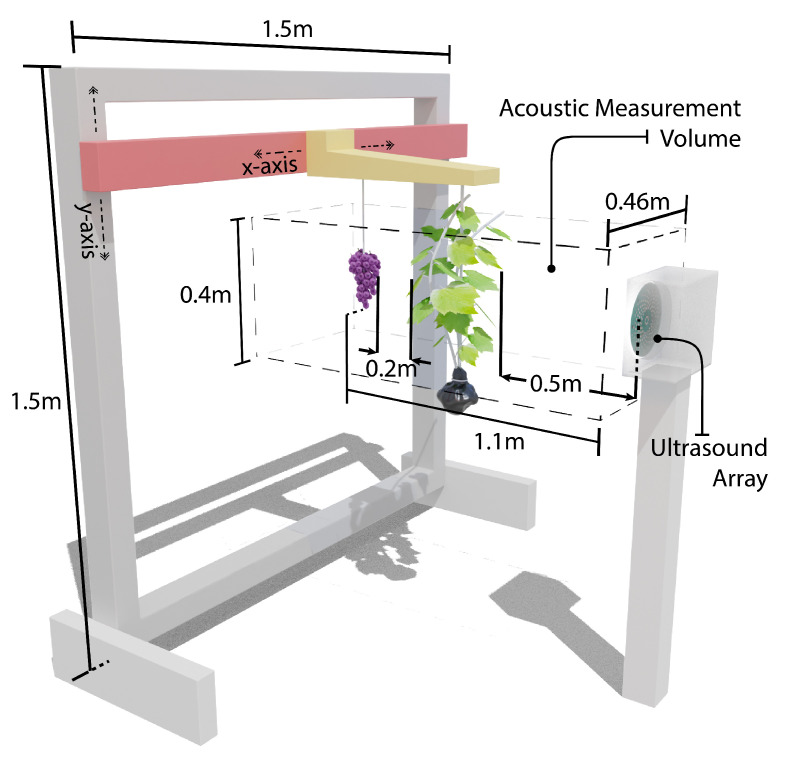
Diagram of the experimental setup showing the grapevine and grape bunch suspended from the CNC machine in front of the ultrasonic array and volume of area where ultrasonic measurements were performed.

The experimental setup and measurement scan volume are illustrated in Figure 7. The ultrasonic transducer was positioned facing the CNC machine at a distance of 1100 mm in front of the grapes. Measurements were made over a 460 × 400 mm wide grid with a spatial separation between ultrasonic measurements of 20 and 50 mm in the *x* and *y* axis respectively. This gave 216 measurement points. Between each ultrasonic measurement, the CNC was paused 3 seconds to allow time for the vine and grapes to stop moving before ultrasonic measurements were made. This measurement procedure was repeated for each of the types of scans described below.

It was anticipated that it would be challenging to differentiate echoes from leaves from that of grape bunches. To try to address this, ultrasonic measurements were therefore made with a fan lightly agitating the leaves, while the heavier grape bunches remained stationary. This agitation could be achieved in the field using a fan or even possibly utilising naturally occurring wind.

The following sets of ultrasonic measurements were therefore made for (a) grapes only with no vine present, (b) both grapes and vine with no fan, and (c) grapes and vine with the fan operating. The fan was was pointed in front of the array and used to lightly agitate the vine leaves. Using a handheld anemometer, the wind-speed at the location of the vine-foliage was measured to be 2.5 m/s. More work is needed in the future to investigate the relationship between air-speed and the resulting agitation performance.

### Measurement of Grape Volume Using Photogrammetry

**Figure 8:**
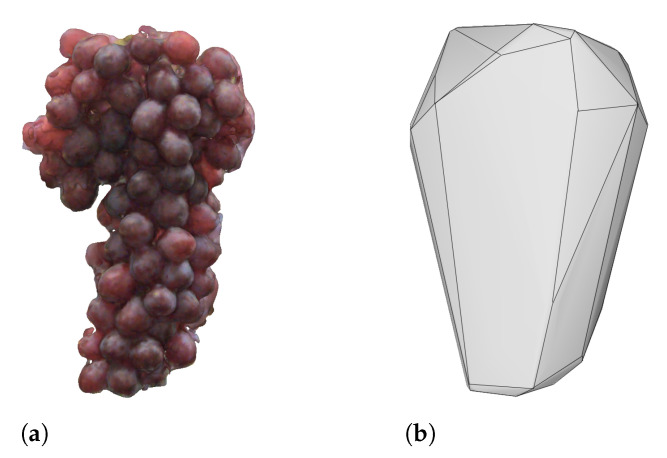
Renders of the 3D scan (**a**) of the grape cluster constructed using photogrammetry and the corresponding convex hull (**b**) created in MeshLab 2020.07.

In Section 5 and Section 6, the ultrasonic measurements are processed to provide an estimate of the volume of the grapes. To provide a comparison (ground truth), the volume of the grapes needed to be measured using an alternative technique. A photogrammetry process was therefore used to construct an accurate 3D scan of the grape cluster. This was achieved by using Agisoft Metashape Professional v1.5.2 to process 30 images captured by a Sony A6300 covering the grape cluster from all sides. The resulting scan can be seen in conjunction with a convex hull approximation in Figure 8.

We have used a convex hull as it offers a representation closer in likeness to the results of this acoustic scan, in that, the concave details of the individual grapes are removed. The convex hull was computed using the convex hull tool in Meshlab 2020.07. The volume of the 3D scan and convex hull are given in Table 2.

## 4. Processing Array Data

### 4.1. Beamforming to Improve Spatial Resolution

An array of ultrasonic sensors can achieve much higher resolution than can be achieved from a single ultrasonic sensor [26,27]. For reception (RX), this was achieved by combining the 12 microphone receiver channel signals into a single channel of data using beamforming.

The echoes from objects were captured by the M=12 microphone ring channels. The record duration was 20 ms which corresponds to N = 4500 samples and a maximum resolvable depth of roughly 3.4 m. The microphone data was stored as a [N×M] matrix x, where the mth column corresponds to the data for the mth microphone ring and is expressed as $x_{m}$.

**Figure 9:**
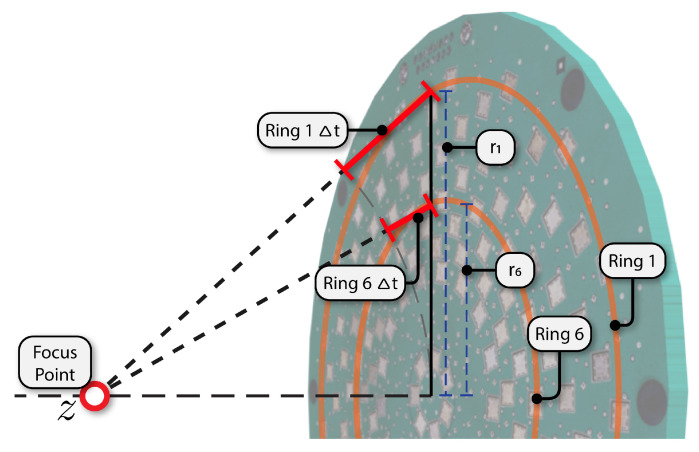
The difference in path length and hence time delay Δt is illustrating in this diagram for two of the array rings when focusing in the near-field at a distance *z* in front of the array.

Figure 9 shows a CAD diagram of the ultrasonic array PCB with two of the microphone rings shown as circles. The time delays required to focus the array in the near-field at a point *z* in front of the array are also illustrated. The reception of the array can be focused at a desired distance *z* along a line normal to the centre of the array using beamforming, see Figure 9. To achieve this, a delay can be calculated for each microphone ring using

(2) $$\Delta t_{m}\left(z\right)=\frac{\sqrt{r_{m}^{2}+z^{2}}-z}{c},$$

where $r_{m}$ is the radius of the mth microphone channel ring given Table 1, and *c* is the speed of sound in air. We can convert this delay to an integer number of samples using

(3) $$\Delta n\left(z\right)=\mathrm{round}\{\Delta t_{m}\left(z\right)\times f_{s}\},$$

where $f_{s}$ is sampling rate.

**Figure 10:**
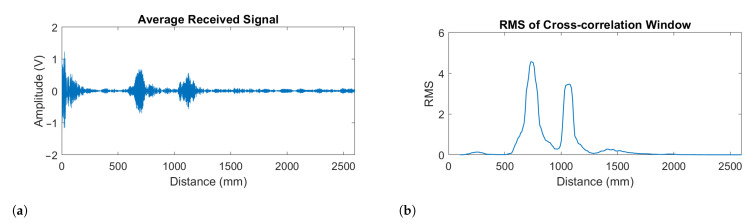
Example plots showing (**a**) the received signal with all the microphone rings averaged, and (**b**) the corresponding windowed RMS (root mean square) representation of the cross-correlated signal.

The [N×M] microphone channel data matrix x can be converted to a [N×1] array of data $\bar{x}$ which is focused at a distance *z* using delay and sum (time domain) beamforming

(4) $$\bar{x}\left[n\right]=\frac{1}{M}\sum_{m=1}^{M}x_{m}\left[n-\Delta n\left(z\right)\right],$$

where *n* is the sample index. See Figure 10a for an example of this summed signal. Note that as *z* becomes large the beamforming delays Δn go to zero. From Equation (4), we can therefore see that averaging all 12 microphone channels (no delays) focuses the array at infinity. This will be referred to here as far-field beamforming. A 40 kHz notch filter was then applied to the resulting signal $\bar{x}$ to remove the ringing at the transducers resonance frequency.

A problem with using time domain beamforming is that the delays that can be applied must be a multiple of the sampling interval, see Equation (3). This can mean that unless the sampling rate is high, the focus may not be accurate. An alternative technique is to use frequency domain beamforming since this does not have this quantisation issue and can therefore be more accurate. Frequency domain beamforming can be achieved by shifting the individual microphone channels using

(5) $${x_{\mathrm{sm}}\left(\omega ,\Delta t_{m}\left(z\right)\right)\;=x_{m}\left(\omega \right)\;e}^{\left\{-i\;\omega \;\Delta t_{m}\left(z\right)\right\}},$$

where *X* is the complex discrete Fourier transform of the recorded signal and ω is the angular frequencies [28]. The phase shifted signal can then be converted back to the time domain using the inverse Fourier transform and summed into a single beamformed channel $\bar{x}$.

There were 20 recordings made at each measurement location of the CNC, giving 20 sets of $\bar{x}$ vectors. The average, μ and variance, $\sigma^{2}$ of these were then calculated element wise for each sample resulting in [N×1] average and variance vectors.

The beamformed signal will contain peaks corresponding to echoes from reflectors in front of the array. The distance to the reflectors can be obtained by converting the time $t_{n}$ when an echo peak occurs in the signal to a distance using

(6) $$d=\frac{t_{n}}{2}\;c,$$

where *c* is the speed of sound. Note that the division of the time by 2 in this equation is due the fact that the echo signal has to travel twice the distance from the array to the object. The speed of sound in air can be approximated as

(7) $$c=c_{o}\;\sqrt{1+\frac{T}{273}}$$

where *T* is the temperature in degrees Celsius and $c_{o}=331.5$ m/s [29]. The ambient temperature was measured using a temperature sensor included as part of the hardware. It was found to be 23 ± 0.25 ${}^{{}^{\circ}}$C giving a speed of sound of 344.8 ± 0.15 m/s. In an outdoors environment, the ambient temperature would be expected to fluctuate more. This would require the air temperature to be monitored closely to allow real-time compensation for the speed of sound on distance measurements.

### 4.2. Cross-Correlation to Improve Depth Resolution

The temporal/depth resolution of the system was improved using cross-correlation [30]. The cross-correlation was calculated using

(8) $$r_{hx}\left[n\right]=\sum_{k=-\infty }^{\infty }\mu \left[k\right]h\left[k-n\right],$$

where h[n] is a filtered version of the signal y[n] applied to the transducers [31]. This filtered the signal to simulate the frequency response of the transducers which is shown in Figure 3. An example of the result of this process can be seen in Figure 10b where the two resolved echoes correspond to the leaves and grapes.

### 4.3. Correction for the Array’s Transmission Being out of Focus

The above cross-correlation technique assumes that the ultrasonic echoes from a point source located directly in front of the array will result (after beamforming and averaging of the received signal) in a signal μ that is a scaled and delayed version of the transmit signal h. However, for a planar array, this is only true if the array is correctly focused (correct beamforming time delays are applied for each transmission and reception array channel). Incorrect focusing of the array will result in signal μ being received from a point reflector that is distorted and not a scaled version of the transmit signal h. This distortion will cause reduced efficiency/errors in the cross-correlation technique.

The reception of the array is able to be focused in postprocessing for any desired distance from the array since each of the 12 microphone rings was sampled using an independent ADC channel. However, for the transmission, this was not possible since the transducer rings were wired in parallel. Even, if the transmission could have been focused (if they had an independent DAC and power amplifier per transducer ring), it would have only been possible to focus at one distance per transmission. Unlike reception, transmission focusing cannot be done in postprocessing. This means that multiple transmissions would be required to allow focusing at a range of distances in the scan volume.

To overcome these issues, a technique was therefore developed to correct for this near-field distortion effect of the transmission in post process. Rather than using the signal y(t) that was applied to the transducers for cross-correlation, a new distorted version of this transmit signal was simulated using

(9) $$\bar{y}\left[n\right]=\frac{1}{M}\sum_{m=1}^{M}y\left[n-dn\left(z\right)\right].$$

This distorted simulated signal was then bandpass filtered by the frequency response of the transducers to give h[n] and used with Equation (8), the beamformed and averaged reception signal μ, to give the cross-correlation $r_{rh}\left[n\right]$ for any desired imaging distance *z*. For each scan, this process was repeated for a range of distances. We have not been able to find this technique being used before in the literature.

### 4.4. Estimating Volumes of Scattering Objects

**Figure 11:**
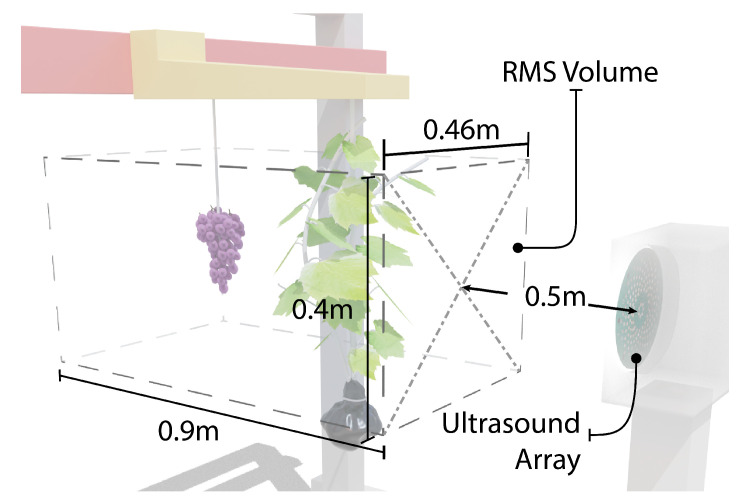
Diagram showing the scan volume used for the RMS processing for grape and leaf detection.

The cross-correlation signal could be plotted as function of distance by converting sample times to distance using Equation (6). A sliding window with a width of 26 samples and a 50% overlap was then used to convert the cross-correlation data to an array of RMS values, where the distance separation between RMS values was 10 mm. This RMS windowing technique was implemented over the scan volume of size 460 × 400 × 900 mm, as shown in Figure 11. Within this volume, 19,224 scan points were defined by dividing the volume up respectively into 24 × 9 × 89 uniformly spaced points.

Isosurfaces are used to visualise the computed volumes using a threshold of 10% of the maximum RMS for each scan. This results in a 3D surface representation of the volume that encompasses all points that have a value at least 10% of the maximum RMS recorded. If this threshold were 0% then the isosurface would represent the entire measurement volume. This threshold was empirically determined to best demonstrate the response of the system. The numerical volume of each isosurface can be naively determined by treating the grid points as voxel cuboids and counting those that are over this threshold within a given region. Each voxel in this measurement corresponds to 20 × 50 × 10 mm = 10 mL. Other techniques for measuring the volume could be investigated in the future such as mean-shift or k-means clustering [32].

## 5. Results for Far-Field Focusing of the Array

**Figure 12:**
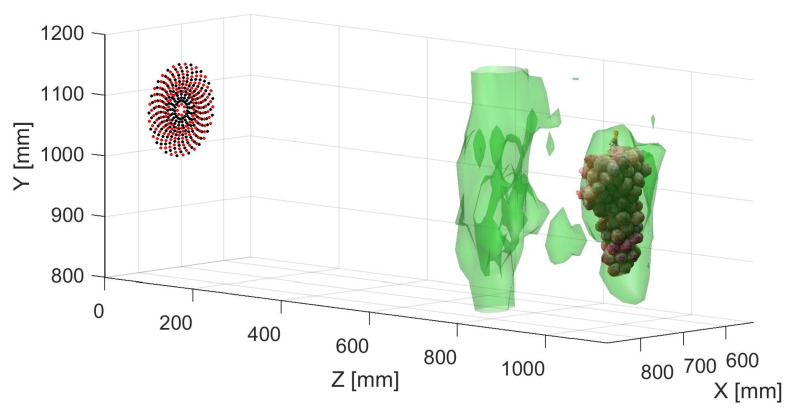
An plot showing an RMS isosurface plot for scans of a grape bunch (at about *z* = 1100 mm) and leaves (at about *z* = 700 mm), where 20 averages were made of the measurement at each position. The 3D photogrammetry scan of the grapes and the microphone (black dots) and transducer (red dots) arrays have been overlaid. It can be seen that the volume of the leaves is significant.

Scans were first made without agitating the grapes with a fan and averaging of repeated samples. An example of a resulting RMS isosurface can be seen in Figure 12. This shows two volumes corresponding to the leaves with the grapes behind. This plot shows that the grapes can be detected behind leaves.

### 5.1. Differentiation of Leaves and Grapes

The measurements described above showed that the grapes and leaves could be detected using ultrasound. However, this technique did not allow one to identify if the reflections were coming from leaves or grapes. We believed that agitating the leaves with a fan might allow this to be achieved. For each position of the CNC machine, 20 recordings were made. The microphone signals from these recordings were averaged (equivalent to far-field beamforming) and the variance obtained.

**Figure 13:**
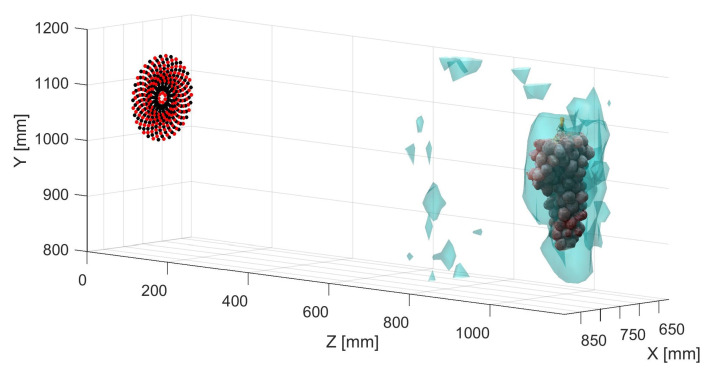
The isosurface plot was achieved using a fan to agitate the leaves and performing filtering of the signal using the average and variance for 20 recordings. When comparing this plot with Figure 12, one can see that this technique allowed one to mainly remove the echoes from the leaves.

Figure 13 shows the resulting isosurface plot after the ultrasonic echo signal had been filtered using average and variance. The movement of the leaves resulted in an increased variance for the the leaves compared to that of the grapes. This technique was able to remove almost all of the signal from the leaves and identify the grapes. A further filter could be added to remove isolated smaller isosurfaces that had an area too small to be expected to be a grapes. Table 3 compares the estimated volumes of the grapes and leaves using these techniques.

## 6. Results for Near-Field Focusing of the Array

The results shown this far present a potential process for the identification of grape clusters in the presence of foliage. However, while promising, the results suggest that more resolution and detail of the canopy can be obtained if the acoustic array had a narrower beamwidth. Although the inherent far-field beamwidth of the array is very narrow, it still diverges at roughly 3.3 degrees. At a distance of 1 m, this equates to a circular cross section of around 58 mm, making it difficult to distinguish between tightly packed objects. Decreasing this beamwidth further would improve the array’s ability to reject reflected sound from nearby objects. Near-field focusing of the array could help improve imaging resolution and hence provide more accurate representation of the scene resulting in a better understanding of the true canopy volume.

**Figure 14:**
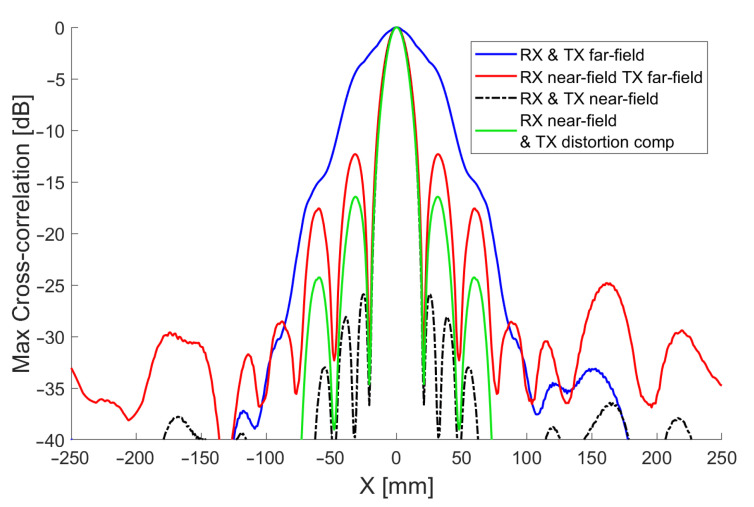
Simulated 60 kHz cross-correlation beam patterns for echoes from a point source reflector located 700 mm from the array employing far-field and near-field beamforming for reception (RX) and transmission (TX). Shown in green is the combined RX near-field and TX distortion compensated beam pattern that was used in this study.

As discussed in Section 4.1, we can achieve near-field focusing of the array using beamforming of the microphone/receiver signal (RX beamforming). With this approach, the microphone receiver array can be focused at a particular distance from the centre of the array, increasing sensitivity at that point and reducing sensitivity to surrounding points. It will also minimise distortion of the signal, which will improve cross-correlation performance. This focusing can be achieved by calculating the phase difference of arrival to each microphone from a sound wave reflected off an object at the focus distance. A corresponding phase shift is then applied to each microphone channel’s recording. The simulated beam patterns shown in Figure 14 indicate that focusing the array in this way could improve the angular resolution substantially. These beam patterns were generated by simulating the sound propagation from each transducer in the array to a reflector situated at a perpendicular distance of 700 mm from the face of the array. The received signal after processing is compared to the transmitted signal using the maximum cross-correlation as discussed in Section 4.2. The resulting maximum correlation for each x position is shown in power form, normalised to 0 dB. The simulation shows a significant reduction in −3 dB beamwidth, from 44 mm to 17 mm, and reduced sidelobes when the correct focus obtained with near-field beamforming is used.

We could extend this process further by applying the same technique to the transmitted signal. As the transducers also have a significant spatial separation, a synchronously transmitted waveform from each ring of transducers, will reach a particular focus distance at slightly different times. This will cause the apparent signal at that point to become distorted. Traditionally beamforming of transmission signal (TX beamforming) would be performed before transmission to compensate for these delays and ensure the signal reaches its target distance undistorted. The result of performing this TX beamforming is shown simulated in Figure 14. It shows a marked improvement in −3dB beamwidth, from 44 mm to 16 mm when using both RX and TX beamforming. Furthermore, sidelobes suppression is significantly improved, showing a 13 dB improvement over just using RX beamforming.

RX beamforming provides a significant improvement to the arrays performance and can be applied to a single recording for all distances in postprocessing. Unfortunately, traditional TX beamforming requires multiple transmissions to cover the entire depth range which makes it largely impractical for our situation. These unique transmissions would take a considerable amount of time to perform and increases the complexity of deploying a real-time system for use in vineyards. Furthermore, it removes the ability to evaluate different focus distances after measurements are conducted. In some situations, it may be beneficial to change the z-axis resolution to get a more detailed view of the scene. Additionally, TX-focusing requires additional hardware in terms of an independent DAC and power amplifier per transmission ring.

To work around these limitations, in Section 4.3, we introduced a novel technique to compensate the cross-correlation for the distortion that affects a transmitted signal when it is not correctly focused. This has the benefit of being computed after capture during postprocessing, in conjunction with RX beamforming, allowing for optimal results at all distances with a single scan. Equation (9) from Section 4.2 describes how the distorted signal at a desired depth can be calculated to then enhance cross-correlation performance. The simulated performance of this technique can be seen in Figure 14. The process results in a substantial improvement over just using RX beamforming. Sidelobes see a further 4 dB of suppression and the −3 dB beamwidth is reduced from 18 to 16 mm. These improvements translate to more granular resolution in the 3D volumetric scans of grape vines. The narrower beamwidth should allow more detail to be captured of the vines and the reduced sidelobes will reduce susceptibility to multipath interference from nearby foliage and other reflectors.

**Figure 15:**
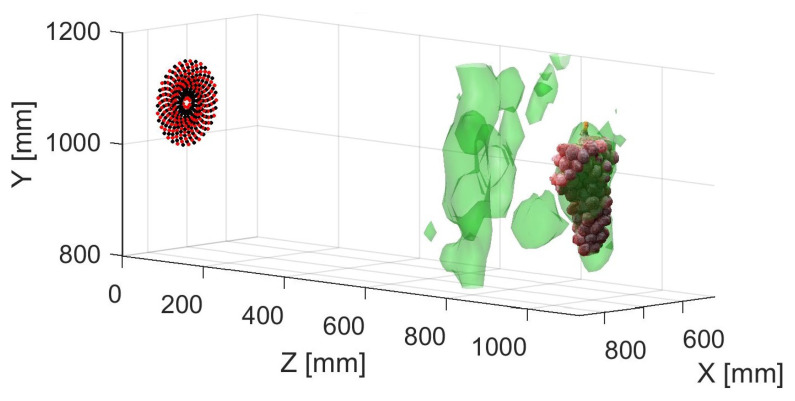
Isosurface visualisation of near-field RX beamformed and TX distortion compensated acoustic scan with averaging of 20 recordings at each scan point. The additional level of detail can be clearly seen in the focused RMS volume.

If we repeat the process used to generate an RMS volume as discussed in Section 4.4, we can compute a comparable volumetric representation using the improved near-field beamforming technique. The resulting RMS volume shown in Figure 15 is presented as an isosurface with the threshold set to 10% of the maximum RMS. A direct comparison can be made to the unfocused scan seen in Figure 12. As can be seen, there is a significant increase in the level of detail in the 3D volume. The volume is less globular and more defined. Increasing observable detail of the structure of the vine canopy could lead to improved vineyard management through more precise knowledge about foliage density and crop loading.

**Figure 16:**
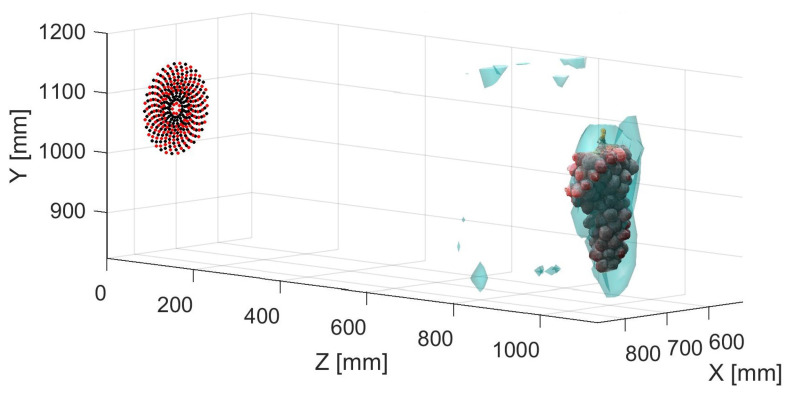
Isosurface visualisation of near-field RX beamformed and TX distortion compensated acoustic scan where echoes from leaves had been mitigated using a fan to agitate the leaves and performing filtering using the average and variance of 20 recordings at each scan point.

The reflections from the leaves can be mitigated in the focused scans using the technique described in Section 5.1. A fan was used to agitate the leaves. Filtering was performed using averaging and variance. Figure 16 shows the resulting isosurface plot. Table 4 compares the grape and foliage volumes obtained using the near-field focused techniques.

## 7. Conclusions and Future Work

This paper presents a novel approach for the detection of grape clusters which are occluded by foliage using an ultrasonic array. It utilises a low frequency ultrasonic chirp transmitted from a highly directional acoustic array. This is the first time that an ultrasonic phased array has been used to analyse canopy structures and the first time ultrasound has been used to visualise grape clusters. The results show that it is possible for low frequency ultrasound to penetrate through leaves and generate echoes from the grapes behind. In addition, the echoes from grapes and leaves can be distinguished by agitating the leaves using a fan and using the variance of multiple recordings as a filter.

We further demonstrate how increased detail in the acoustic volumes can be achieved through near-field focusing the reception of the array using beamforming and cross-correlation defocusing correction techniques. This significantly reduces the beamwidth and increases directionality of the array. The increased level of detail has direct benefit for more accurate canopy estimation and as a result, improved precision viticulture practices.

Improved spatial and depth resolution would also be expected to reduce the overestimation in volume measurement obtained using the ultrasonic measurement. Table 5 compares the percentage overestimation in volume obtained in Table 3 and Table 4 using the ultrasonic methods compared to the volumes of the the 3D photogrammetric scan and the convex hull given in Table 2 which was obtained using photogrammetry. Here we can see that the use of near-field focusing techniques with averaging reduced the ultrasonic measured overestimation in grape volume from 222% to 112% compared to the photogrammetry scan or from 56% to 2.5% compared to the convex hull of this scan. More work is needed to investigate how these results would vary with different volume estimation techniques from that used in this work or using a finer measurement grid spacing with the CNC.

It is worth noting that while it may be possible to determine true volume estimates using the acoustic techniques mentioned in this paper, the presented numerical volumes should only be considered as relative comparisons of the effect of each stage of the process. The establishment of an accurate relationship between acoustic volume and true cluster volume will require further study with a range of different grape clusters and foliage conditions. However, it should be noted that accurate measurement of the occluded grape volume using ultrasound is not necessarily essential. For example, it could potentially provide improved estimates of the proportion of occluded grapes to enhance yield estimates obtained using other methods such as computer vision techniques.

The process presented in this paper represents a significant improvement over the current state of the art ultrasonic methods for vine canopy assessment. The increased achievable detail will have a direct benefit for 3D volume estimation of vine canopies as well as improved ability to resolve potential grape clusters. These improvements should enable viticulturists to implement advanced precision viticulture techniques such as crop thinning, precise variable rate applications, and selective harvesting. We also anticipate that the techniques used will have applications beyond viticulture to other areas of horticulture.

### Future Work

The lab results presented in this paper show promising initial results. However, field trials are needed to investigate how this system performs in a vineyard environment with different grapes varieties. The performance of the system needs to be investigated further with more leaves, grapes in closer proximity to the leaves, and occluding objects such as vine stems, trunks, and trellis materials. Solid obstacles such as trunks and trellis would not be disturbed by the agitation. This could be addressed by using a fusion of ultrasound and computer vision to assist in identifying these objects. Traditional computer vision techniques can be used to label visible areas of the ultrasonic scan such as vine stems, trunks, and other solid objects. This could be extended further to develop an unsupervised machine learning process to directly classify regions of the acoustic recordings.

The effect of the presence of neighbouring grape clusters also needs to be investigated since they are likely to appear as a single larger cluster with the current processing and hardware. Scanning from different directions may provide improved ability to see behind solid objects or differentiate grape bunches which would otherwise be hidden by another cluster. Work is also needed to identify how early in the season this ultrasonic technique can be used to identify grapes bunch clusters and the relationship between the acoustic scan output and the true cluster weight.

Near-field focusing required additional processing overhead compared with far-field beamforming. One approach to address this may be to preclassify regions of the signal that contain significant reflected components and only perform beamforming on these regions. This could be assisted by incorporate a 3D depth cameras to provide additional information on where processing should be performed. Additionally, as each measurement location is independent of the others, simple parallelization techniques can be used to vastly improve processing times. In addition, improved resolution could be achieved by modifying the hardware so that the transmission could be focused in the near-field.

The array used in this study featured a very narrow beamwidth and could only image directly in front of the array. It used a highly accurate CNC machine to generate the 3D acoustic scans of the grapes. Although beyond the scope of this project, we believe we can enhance the hardware further to increase its practical use within a vineyard by reducing the scan time and remove the need for the CNC machine. Accurate tracking of the position of the array without the use of a CNC could be achieved using techniques such as a fusion of differential GPS and optical pose estimation.

If the array was redeveloped for large scale field trials, different transducers are likely to be used which may have different optimal transmitted signal. Therefore, it would be beneficial to further investigate the effect of transmitted waveform on the resulting scan and their resilience to sources of interference such as multipath reflections. Furthermore, given the significant physical differences between grape clusters and vine foliage, it may be possible to identify unique frequencies of absorption or reflection for each potentially making it possible to classify directly from the recorded waveforms.

## Figures and Tables

**Table 1:** Radii of microphone and transducer rings.

Ring Number	Microphone (mm)	Transducer (mm)
1	15.0	9.0
2	20.3	31.0
3	25.7	36.4
4	31.0	41.8
5	36.4	47.3
6	41.8	52.8
7	47.3	58.3
8	52.8	63.8
9	58.3	69.4
10	63.8	75.0
11	69.4	
12	75.0	

**Table 2:** Grape volume measured using photogrammetry.

	3D Scan	Convex Hull
Grape volume [mL]	580	1200

**Table 3:** Grape and foliage volume estimates using ultrasonic far-field array focusing.

	Static	Averaged	Variance Filtered
	(fan off)	(fan on)	(fan on)
Grape volume [mL]	1660	1870	3500
Leaf volume [mL]	8510	3890	870

**Table 4:** Grape and foliage volume estimates obtained using near-field RX and TX focusing of the array for different types of scans.

	Averaged	Variance Filtered
Grape volume [mL]	1230	1320
Leaf volume [mL]	3470	280

**Table 5:** Percentage overestimation of the measured grape volume obtained using near-field and far-field focusing compared to the grape volume obtained using photogrammetry 3D scan (580 mL) and convex hull (1200 mL).

	Far-Field Focusing			Near-Field Focusing	
	Static	Average	Filtered	Averaged	Filtered
Photogrammetry	186%	222%	503%	112%	128%
Convex Hull	38%	56%	192%	2.5%	10%

## References

[1] Matese A., Gennaro S.F.D. (2015). Technology in precision viticulture: A state of the art review. Int. J. Wine Res..

[2] Bramley R., Proffitt A. (1999). Managing variability in viticultural production. Grapegrow. Winemak..

[3] Nuske S., Achar S., Bates T., Narasimhan S., Singh S. (2011). Yield estimation in vineyards by visual grape detection. Proceedings of the 2011 IEEE/RSJ International Conference on Intelligent Robots and Systems (IROS).

[4] Nuske S., Gupta K., Narasimhan S., Singh S. (2014). Modeling and calibrating visual yield estimates in vineyards. Field and Service Robotics.

[5] Mirbod O., Yoder L., Nuske S. (2016). Automated measurement of berry size in images. IFAC-PapersOnLine.

[6] Herrero-Huerta M., González-Aguilera D., Rodriguez-Gonzalvez P., Hernández-López D. (2015). Vineyard yield estimation by automatic 3D bunch modelling in field conditions. Comput. Electron. Agric..

[7] Dey D., Mummert L., Sukthankar R. (2012). Classification of plant structures from uncalibrated image sequences. Proceedings of the 2012 IEEE Workshop on Applications of Computer Vision (WACV).

[8] Eccleston K.W., Platt I.G., Tan A.E.-C. (2018). SAR for grape bunch detection in vineyards. Proceedings of the Microwave Symposium (AMS), 2018.

[9] Gil E., Escola A., Rosell J., Planas S., Val L. (2007). Variable rate application of plant protection products in vineyard using ultrasonic sensors. Crop. Prot..

[10] Llorens J., Gil E., Llop J., Escola A. (2010). Variable rate dosing in precision viticulture: Use of electronic devices to improve application efficiency. Crop. Prot..

[11] Mazzetto F., Calcante A., Mena A., Vercesi A. (2010). Integration of optical and analogue sensors for monitoring canopy health and vigour in precision viticulture. Precis. Agric..

[12] Tumbo S., Salyani M., Whitney J.D., Wheaton T., Miller W. (2002). Investigation of laser and ultrasonic ranging sensors for measurements of citrus canopy volume. Appl. Eng. Agric..

[13] Palacin J., Pallejà T., Tresanchez M., Sanz R., Llorens J., Ribes-Dasi M., Masip J., Arno J., Escola A., Rosell J.R. (2007). Real-time tree-foliage surface estimation using a ground laser scanner. IEEE Trans. Instrum. Meas..

[14] Llorens J., Gil E., Llop J. (2011). Ultrasonic and lidar sensors for electronic canopy characterization in vineyards: Advances to improve pesticide application methods. Sensors.

[15] Palleja T., Landers A.J. (2015). Real time canopy density estimation using ultrasonic envelope signals in the orchard and vineyard. Comput. Electron. Agric..

[16] Palleja T., Landers A.J. (2017). Real time canopy density validation using ultrasonic envelope signals and point quadrat analysis. Comput. Electron. Agric..

[17] Kazys R.J., Vilpisauskas A., Sestoke J. (2018). Application of air-coupled ultrasonic arrays for excitation of a slow antisymmetric lamb wave. Sensors.

[18] Allevato G., Hinrichs J., Rutsch M., Adler J., Jäger A., Pesavento M., Kupnik M. (2020). Real-time 3D imaging using an air-coupled ultrasonic phased-array. IEEE Transactions on Ultrasonics, Ferroelectrics, and Frequency Control.

[19] Legg M., Bradley S. (2019). Ultrasonic arrays for remote sensing of pasture biomass. Remote Sens..

[20] Legg M., Bradley S. (2019). Ultrasonic proximal sensing of pasture biomass. Remote Sens..

[21] Almqvist M., Holm A., Persson H.W., Lindström K. (2000). Characterization of air-coupled ultrasound transducers in the frequency range 40 kHz-2 MHz using light diffraction tomography. Ultrasonics.

[22] Parr B., Legg M., Alam F., Bradley S. Acoustic identification of grape clusters occluded by foliage. Proceedings of the Sensors and Applications Symposium (SAS 2020).

[23] DT9836 Series: High-Speed Simultaneous USB Devices with BNC. https://www.mccdaq.com/Products/Multifunction-DAQ/DT9836.

[24] Gan T.H., Hutchins D.A., Billson D.R., Schindel D.W. (2001). The use of broadband acoustic transducers and pulse-compression techniques for air-coupled ultrasonic imaging. Ultrasonics.

[25] ISO 9613-1:1993 (1993). Acoustics—Attenuation of Sound During Propagation Outdoors—Part 1: Calculation of the Absorption of Sound by the Atmosphere.

[26] Yuan W., Zhou T., Jiajun S., Du W., Wei B., Wang T. (2020). Correction method for magnitude and phase variations in acoustic arrays based on focused beamforming. IEEE Trans. Instrum. Meas..

[27] Camacho J., Martinez O., Parrilla M., Mateos R., Fritsch C. (2010). A strict-time distributed architecture for digital beamforming of ultrasound signals. IEEE Trans. Instrum. Meas..

[28] Legg M., Bradley S. (2014). Automatic 3D scanning surface generation for microphone array acoustic imaging. Appl. Acoust..

[29] Oxford Reference: Speed of Sound. www.oxfordreference.com/view/10.1093/oi/authority.20110803100522606.

[30] Queiros R., Alegria F.C., Girao P.S., Serra A.C. (2010). Cross-correlation and sine-fitting techniques for high-resolution ultrasonic ranging. IEEE Trans. Instrum. Meas..

[31] Proakis J., Manolakis D. (1996). Digital Signal Processing: Principles, Algorithms, and Applications.

[32] Ximin Z., Wanggen W., Li X., Junxing M. (2014). Mean shift clustering segmentation and ransac simplification of color point cloud. Proceedings of the 2014 International Conference on Audio, Language and Image Processing (ICALIP).

